# Chicago Medical Society

**Published:** 1879-05

**Authors:** 


CHICAGO MEDICAL SOCIETY, Regular Meeting April 21st, Dr. Andrews, president, in the chair.

The president read a paper on "The Treatment of Hæmorrhoids by Injection," which was substantially the same as his article on the same subject in the present number of the JOURNAL. The paper being before the society for discussion, DR. BYFORD, after commending it highly, inquired concerning the use of other strong acids beside the carbolic applied locally, and as formerly employed. He had often found them effectual, nitric acid particularly, and usually comparatively painless.

DR. ENGERT observed that she had used the ungentum hydrargyri ammoniati, U. S. P., in several cases with excellent effect.

DR. INGALS had enjoyed listening to the paper, and was especially glad to see that Dr. Andrews had not hesitated to go outside of the regular professional ranks, and gather information from every available source. Many of the charlatans were reckless or ignorant enough to resort to practices which we would not dare to employ; and yet we ought, in justice, to profit by their experience. He also inquired concerning the local use of iodine for piles; he had had no experience with it, but thought that, on theoretical grounds, it might be used with advantage.

DR. FENN said that he had been in the habit of treating many of these cases more as medical cases than as surgical; that is treating the digestive organs which were often at fault, and endeavoring to perfect the processes of digestion and elimination—which was often of prime importance. But he should be more ready now than ever to resort to radical means of relief by the method of injection.

DR. STEELE asked if the writer of the paper had had any experience in rupturing the sphincter ani.

DR. PAOLI thought that by many of the means of treatment strictures of the rectum had been produced from the cicatrization following removal of tissue. He insisted that in persons of intemperate habits or "high-livers" there was a necessity that they should adopt a temperate life and follow simple hygienic rules. He also said that he had given relief by using suppositories containing iodoform.

DR. HAYES said he understood that certain of the quacks used a little clamp about the base of the pile to obstruct the circulation before injecting it. Knowing the effect of ergot he had more than once used a simple gelatine coated 3-grain pill of ergotine as a suppository, and this with excellent result.

DR. BOGUE had used injections of 95 per cent. carbolic acid, pure, in six cases, injecting from .03 to 0.40. In five cases the results were certainly beneficial; in the other case uncertain. The pain was inconsiderable in all but one case.

DR. TILLEY stated that Richardson, of London, had been using the sodium ethylate for destruction of vascular tumors of various kinds, and he inquired whether Dr. Andrews had used it for destruction of piles.

In reply to the various queries, DR. ANDREWS stated with regard to the use of strong acids, that there was much contradiction among authors concerning their effectiveness and painlessness. In his opinion, when a pile was uninflamed, nitric acid was effectual and simple; but in proportion as the pile became thickened and developed sensory nerves the acid caused more distress. He had heard of the use of iodine, but was unable to give any definite information concerning it, though he had little doubt that it might be of service. He had never tried to rupture the sphincter in these cases. In one of his early cases he had removed too much tissue and had seen a stricture of the rectum as the result; and he would give a general caution against the removal of too much of the base of the pile, whether it were crushed, snipped off or cauterized. He had not used the sodium ethylate because he had not thought it any better than to use the caustic alkali itself.

Lastly, when promptness of result was desirable on account of sympathetic bladder or uterine inflammation, he thought the method by injections would be too slow, and some more rapid one must be adopted; but when the piles were above the anus, he thought the method of repeated, small, diluted injections the most preferable of any.

